# Safety of Triage Self-assessment Using a Symptom Assessment App for Walk-in Patients in the Emergency Care Setting: Observational Prospective Cross-sectional Study

**DOI:** 10.2196/32340

**Published:** 2022-03-28

**Authors:** Fabienne Cotte, Tobias Mueller, Stephen Gilbert, Bibiana Blümke, Jan Multmeier, Martin Christian Hirsch, Paul Wicks, Joseph Wolanski, Darja Tutschkow, Carmen Schade Brittinger, Lars Timmermann, Andreas Jerrentrup

**Affiliations:** 1 Charité Universitäsmedizin Berlin Berlin Germany; 2 Department of Emergency Medicine, University Clinic Marburg, Philipps-University Marburg Germany; 3 Ada Health GmbH Berlin Germany; 4 Center for Unknown and Rare Diseases, UKGM GmbH, University Clinic Marburg, Philipps-University Marburg Germany; 5 Else Kröner Fresenius Center for Digital Health Faculty of Medicine Carl Gustav Carus Technische Universität Dresden Dresden Germany; 6 Institute of Artificial Intelligence, Philipps-University Marburg Marburg Germany; 7 Coordinating Center for Clinical Trials, Philipps University Marburg, Marburg, Germany Marburg Germany; 8 Department of Neurology, University Hospital of Marburg Marburg Germany

**Keywords:** symptom checker, emergency medicine, app, triage, safety, innovative, eHealth, artificial intelligence

## Abstract

**Background:**

Increasing use of emergency departments (EDs) by patients with low urgency, combined with limited availability of medical staff, results in extended waiting times and delayed care. Technological approaches could possibly increase efficiency by providing urgency advice and symptom assessments.

**Objective:**

The purpose of this study is to evaluate the safety of urgency advice provided by a symptom assessment app, Ada, in an ED.

**Methods:**

The study was conducted at the interdisciplinary ED of Marburg University Hospital, with data collection performed between August 2019 and March 2020. This study had a single-center cross-sectional prospective observational design and included 378 patients. The app’s urgency recommendation was compared with an established triage concept (Manchester Triage System [MTS]), including patients from the lower 3 MTS categories only. For all patients who were undertriaged, an expert physician panel assessed the case to detect potential avoidable hazardous situations (AHSs).

**Results:**

Of 378 participants, 344 (91%) were triaged the same or more conservatively and 34 (8.9%) were undertriaged by the app. Of the 378 patients, 14 (3.7%) had received safe advice determined by the expert panel and 20 (5.3%) were considered to be potential AHS. Therefore, the assessment could be considered safe in 94.7% (358/378) of the patients when compared with the MTS assessment. From the 3 lowest MTS categories, 43.4% (164/378) of patients were not considered as emergency cases by the app, but could have been safely treated by a general practitioner or would not have required a physician consultation at all.

**Conclusions:**

The app provided urgency advice after patient *self-triage* that has a high rate of safety, a *rate of undertriage*, and a *rate of triage with potential to be an AHS,* equivalent to telephone triage by health care professionals while still being more conservative than direct ED triage. A large proportion of patients in the ED were not considered as emergency cases, which could possibly relieve ED burden if used at home. Further research should be conducted in the at-home setting to evaluate this hypothesis.

**Trial Registration:**

German Clinical Trial Registration DRKS00024909; https://www.drks.de/drks_web/navigate.do? navigationId=trial.HTML&TRIAL_ID=DRKS00024909

## Introduction

### Background

The need for acute medical care in emergency departments (EDs) and primary care clinics has become increasingly important from medical and health policy perspectives [1-3]. Partially owing to an aging population and difficulty in accessing other care options, an increasing number of patients with chronic conditions and people with general medical illnesses present in EDs [4]. More than 50% of patients attending an ED stated that they considered their level of treatment urgency as low [5], and studies have shown how challenging it is for patients to assess their own medical urgency level [6-9]. Apart from extended waiting times and patient dissatisfaction, crowded EDs are associated with several risks such as delayed care, persisting pain, poor outcomes, and increased mortality [10]. Timely assessment is increasingly a major problem in terms of staffing and organization in both large and small hospitals. An additional digital system could be of much help here. To address this, we explored whether a digital patient triage solution could provide meaningful assistance to the patient in pretriaging their current health problem, guiding patients with urgency to the ED and others to alternative appropriate care providers including urgent care centers, general practitioners (GPs), or even pharmacies or self-care.

Currently, in an international context, there is no established system of remote *pretriage* or urgency advice, which patients can use before visiting an ED, although a number of solutions have been proposed including telephone triage and video-assisted triage through health care apps [11-13].

The symptom assessment class of home-use health care apps (sometimes known as symptom checkers) [14] has the potential to provide useful information for patients on disposition (ie, the urgency of care-seeking and indicating the appropriate type of health care provider to contact) and to increase the efficiency of the medical workflow through hand over of information on symptoms, history, and risk factors. Individual apps within this class differ in their intended purpose; for example, some can only be used for a narrow range of conditions, age groups, or health care settings [15-20]. One of these symptom assessment apps (SAAs) is Ada, an app designed to be used at home, in which patients enter their risk factors and most troubling symptoms. On the basis of this information, an adaptive question flow is generated using a large medical knowledge database and complex Bayesian networks. A report lists the denied and affirmed symptoms, up to 5 suggestions on conditions including their probability, and an overall urgency assessment to provide the user with information about possible causes for their symptoms and the next steps to consider.

Although vignette studies testing SAAs have been conducted [21-24], not many studies have explored them in a prospective ED setting, which is another area of interest for such apps besides the at-home setting. Barriga et al [25] compared ED physicians’ diagnoses with those from an app; however, they excluded the patient from their analysis if the physician’s diagnosis was not modeled in the app’s system. In addition, a retrospective study explored triage and diagnostic accuracy of 5 SAAs for patients presenting in the ED with HIV or hepatitis C [16]. A further study has examined triage acuity of a web-based SAA in a prehospital setting, but without comparing the data with a gold standard [26]. A recently published study compared the National Health Systems 111 telephone triage system with ED triage (for those patients attending the ED) and showed a high proportion of mistriaged cases [27].

### Objectives

The aim of this study is to prospectively evaluate the urgency advice provided by an SAA (Ada) to examine its extensibility to the ED waiting room triage.

In an observational approach, the safety of the app’s urgency advice in a large German university hospital ED is assessed by comparing the app’s urgency advice levels with the assignments by a trained health care professional (HCP) using a validated triage algorithm (Manchester Triage System [MTS]). An expert physician panel evaluated all the cases of the app’s undertriaged advice. We investigated the hypothesis that the urgency advice provided by the app to patients in the ED waiting room would be similar to triage by HCPs in terms of safety of advice.

## Methods

### Study Population, Setting, and Procedure

The study was conducted at the interdisciplinary ED of Marburg University Hospital, which is attended by approximately 48,000 patients per year, with data collection performed between August 2019 and March 2020. The completed Strengthening the Reporting of Observational Studies in Epidemiology checklist is included in (Table S1 in Multimedia Appendix 1). Written informed consent was obtained from all patients before entering the study. Sample size calculation was performed by the Coordinating Centre for Clinical Trials, Marburg.

Patients were triaged by a triage nurse, following the usual workflow and using the MTS implemented through a computerized decision support system. MTS maps the patient’s presenting complaint to one of 52 flowchart diagrams. After checking the key discriminators for each of these flowcharts, the MTS groups patients into one of 5 urgency categories [28]. Each category has been assigned a maximum time in which the patient has to be examined by a physician, ranging from red (0 minutes waiting time) to blue (120 minutes waiting time in the German version of MTS). Patients grouped into the two highest triage levels, red and orange (maximum of 10 minutes waiting time), were excluded from the study because in this initial observational study, we did not consider it safe and feasible for these patients, who are not the current target population of the app, to complete enrollment and conduct a self-assessment in their available waiting time.

All German-speaking walk-in patients aged ≥18 years attending the ED and triaged yellow, green, or blue were eligible to be included in the study. No department was excluded from the study. All patients who met these criteria were enrolled during the working hours of the assistant in charge of the study. Patients who were already called for examination before being approached or who had left the ED before being examined by medical staff were excluded from the study. The recruitment was performed by the study assistant in the waiting room after ED staff triage. After consent was obtained, patients participated in an assessment on a study iPad prepared with an adapted version of the app (study ID was used instead of a name; report was not shown to the patient after use as the study had an observational design, and to be compatible with this, to prevent information from the report from being passed on to the attending physician and potentially influencing the physicians’ decision and patient outcomes). The study assistant did not offer any content-related assistance, for example, explanations of terms, but only helped with the technical operation. The assessment report was accessible to study staff only.

The patients entered factors such as sex, age, and specific risk factors (hypertension, diabetes, smoking, and pregnancy), followed by their most troubling symptoms, which were the reasons for their ED visit. The app then proceeded through an adaptive question flow, asking the essential next questions to lead to the optimal condition suggestions and urgency advice (on an 8-level scale; Table 1). Then, the patients proceeded to usual care without seeing the app output.

**Table 1:** App grading of urgency recommendations.

Urgency assessment level	Short description of advice level	Recommended next steps
1	Call ambulance	May require emergency care; if the patient considers this to be an emergency, calling an ambulance is advised.
2	Emergency care	May require emergency care; if the patient considers this to be an emergency, they should immediately visit an emergency department.
3	Primary care within 4 hours	May require urgent medical care; the patient is advised to see a primary care physician within the next 4 hours.
4	Primary care within same day	May require prompt medical care; the patient is advised to see a primary care physician, ideally on the same day.
5	Primary care within 2-3 days	No urgent medical care is required; the patient is advised to see a primary care physician, ideally in the next couple of days.
6	Primary care within 2-3 weeks	No urgent medical care is required; the patient is advised to see a primary care physician in a routine appointment.
7	Self-care or pharmacy	No medical consultation is needed; the patient can probably manage symptoms safely at home, and possibly, it could be helpful to consult a pharmacist.
8	Self-care	No medical consultation is needed; the patient can probably manage symptoms safely at home.

### Ethics Approval

This study was approved by the Philipps University Marburg Ethics Committee for the Department of Medicine (133/18).

### Study Design

This study had a single-center cross-sectional prospective observational design to evaluate the safety of urgency advice given by the app. To assess safety and identify all cases where a less conservative advice level could have the potential to harm the patient’s health, we used the approach reported by Meer et al [9] via a physician panel who adjudicated on potential avoidable hazardous situations (AHSs). An AHS is defined as a health-damaging situation that is preventable through timely medical intervention.

The MTS score given by the triage nurse in the ED was compared with the advice level given by the SAA. This comparison was conducted using a predefined mapping (Table S2 in Multimedia Appendix 1), which has three categories: (1) exact match of the recommendations, (2) higher triage recommendation than MTS, and (3) lower urgency than MTS. Patients whose advice level from the SAA was higher than or matching with the MTS score were considered to have been safely triaged. For other patients whose advice level was lower than the MTS score, all case information was collected and reviewed by a panel of physicians. The panel members had no connection to the study center or study team and had a minimum of 9 years of clinical experience and different specialties: a GP and active emergency physician, a specialist in internal medicine, and the chief physician of the ED at a large hospital. The panel considered all the clinical information included in the physicians’ reports and collected by the SAA. Each panel physician individually checked all the cases, assessed the urgency, and, from his point of view, selected the most appropriate advice level without being made aware of the MTS score or the app’s advice level. This was later compared with the actual urgency advice provided by the app for an additional blinded comparison.

Then, each panel physician saw the MTS score and the app’s advice level to adjudicate whether the app’s advice would have been health-damaging if the patient had used the app at home and followed the provided advice (categories: *unlikely, rather unlikely, rather likely,* and *likely health-damaging*). The panel members were asked to record a brief justification for each of their decisions (Table S3 in Multimedia Appendix 1). In a videoconference, the panel members discussed all the cases in which at least one of them chose the categories *likely* or *rather likely* health-damaging. A panel decision for these cases was reached by majority voting (ie, potential to be an AHS).

### Data Collection and Analysis

All data entered by the participants were stored electronically and on paper and entered manually by a study staff member into a database created and managed by the Coordinating Centre for Clinical Trials in Marburg. Clinical data were obtained from the hospital information system (Dedalus ORBIS).

To determine the correspondence of the urgency assessment of the app and the triage nurse, Cohen *κ* coefficient [29,30], including the weighted, prevalence, and bias-adjusted Cohen *κ* coefficient [31], was calculated using R (version 3.6.1; R Foundation for Statistical Computing). This is a classical matching measure but designed for quadratic contingency tables. Therefore, the app’s categories were combined into 3 categories according to content (Table S2 in Multimedia Appendix 1) and assigned to the 3 MTS categories, respectively. In this analysis, the MTS triage was regarded as the reference standard. To assess the advice’s safety, we estimated the probability that the urgency assessment of the app was less conservative than the reference standard. We calculated Fleiss *κ* to assess the interrater agreement.

In addition, the chi-square test was performed to determine whether the distribution of the assessments of urgency differs between the ED medical departments [32]. Missing data were described per analysis, and participants were not excluded for missing data.

## Results

### Patient Selection

In this single-center cross-sectional prospective observational study, patients were enrolled between August 2019 and March 2020. A total of 544 patients were estimated to be enrolled using Cohen *κ* with a 2-sided 95% CI and a width of 0.1 units (p0=0.80; Cohen *κ*=0.70). Owing to the COVID-19 pandemic, the study had to be terminated prematurely before reaching the calculated 554 patients. Early in the pandemic, additional staff, including clinical study personnel, were not permitted to work in the ED to prevent further endangerment of patients and clinical staff.

**Figure 1:**
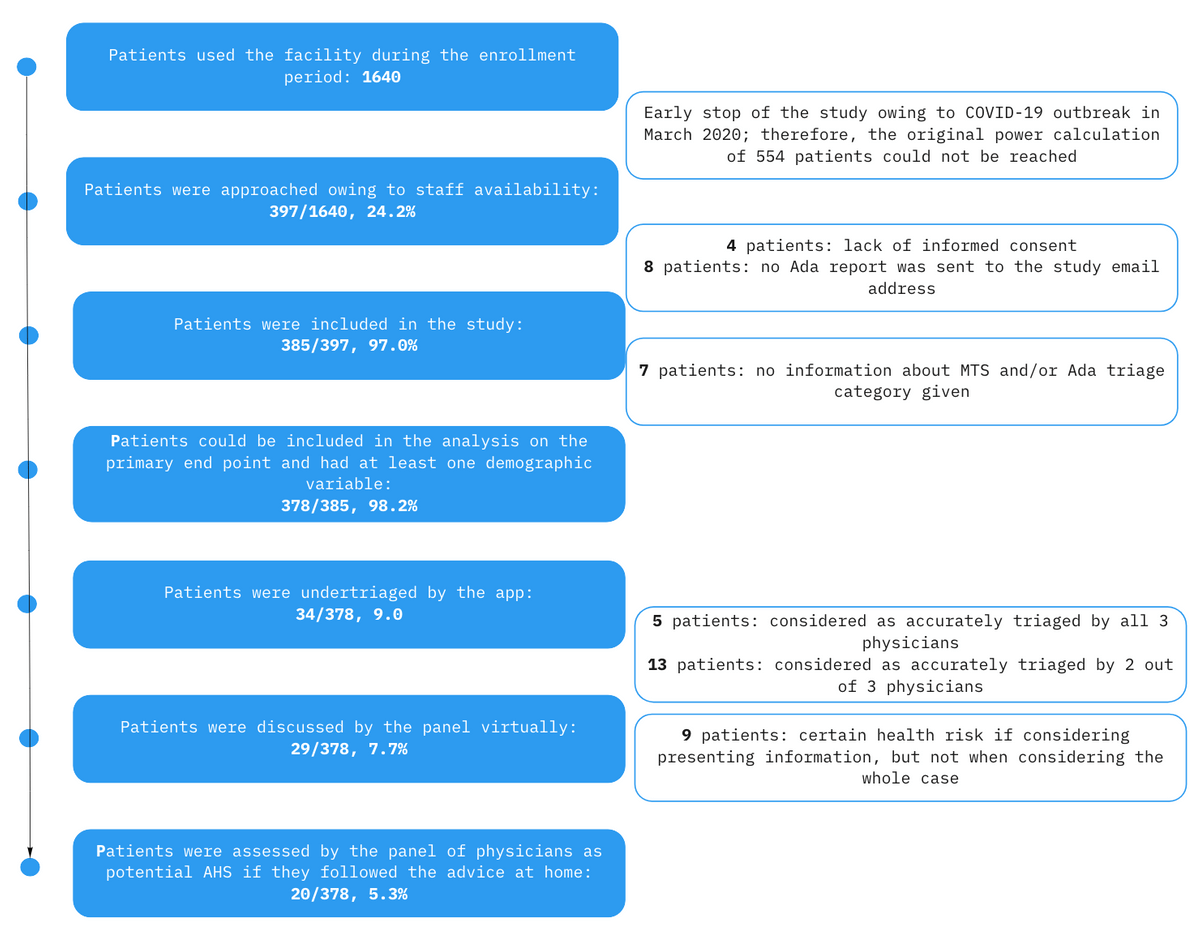
Flowchart of patient recruitment. AHS: avoidable hazardous situation; MTS: Manchester Triage System.

During the study period, 1640 patients who met the eligibility criteria used the facility. Owing to staff availability, only 24.21% (397/1640) of them could be approached, and all of them agreed to participate. Of the 397 patients, 4 (1%) were excluded because informed consent was not complete and 8 (2%) were excluded because of technical problems (report was not sent from the app to the study email address). Therefore, 96.9% (385/397) of the patients were included in the study, of whom 98.2% (378/385) provided enough information to analyze the primary endpoint. For the 1.8% (7/385) excluded patients, either the MTS or the app’s triage level was missing. See Figure 1 for the flowchart of patient recruitment and Table 2 for patient characteristics. The raw data are available in Multimedia Appendix 2.

**Table 2:** Patient characteristics.

Characteristic		Value, n (%)
**Age (years; n=377)**		
	18-29	93 (24.7)
	30-39	59 (15.6)
	40-49	58 (15.4)
	50-59	77 (20.4)
	60-69	55 (14.6)
	70-79	28 (7.4)
	80-89	6 (1.6)
	90-99	1 (0.3)
**Sex (n=377)**		
	Men	215 (57)
	Women	162 (43)
**Location of presenting symptom (N=378)**		
	Infection or feeling generally unwell	6 (1.6)
	Pathological laboratory results	9 (2.4)
	Paresthesia	25 (6.6)
	Digestive	47 (12.4)
	Chest, heart, or lungs	30 (7.9)
	Face: eye, ear, nose, throat, or teeth problem	40 (10.6)
	Head	44 (11.6)
	Upper extremity	59 (15.6)
	Lower extremity	77 (20.4)
	Genitourinary problems	6 (1.6)
	Neck or back	18 (4.8)
	Skin	5 (1.3)
	Other	5 (1.3)
	Missing	7 (1.9)
**Departments (N=378)**		
	Orthopedics and trauma	164 (43.4)
	Internal medicine	102 (26.9)
	Neurology	72 (19)
	Other	40 (10.6)

### Patient Characteristics

The mean age of participants was 46 (SD 17.54; median 46) years. In all, 43.4% (164/378) of the patients were aged ≥50 years and 57% (215/377) of the patients were men. The most common presenting symptom was extremity pain (136/378, 35.9%) followed by gastrointestinal symptoms such as abdominal pain, nausea, or change of bowel movement (47/378, 12.4%). Of the 378 participants, 44 (11.6%) participants presented at the ED with headache or vertigo. Of all participants, 43.4% (164/378) were allocated to the orthopedics and trauma department, 26.9% (102/378) to internal medicine department, and 19% (72/378) to neurology department. Totally, 10.6% (40/378) of included patients were examined at and treated by other departments. When comparing the data from this study with data from a study focusing on patient characteristics in an ED of a German university hospital over the period of a year in 2019, we could see that the mean age of their patient population was 47 (SD 24; median 47, range 0-106) years, which was similar to that reported in this study (mean 46, SD 17.54 years; range 18-94 years) [33]. This study reported fewer female patients (162/377, 43%) than the previous study (48%). When only considering the lower 3 MTS categories, the study reported 39.8% of patients classified as MTS 3, 41.4% of patients as MTS 4, and 4% of patients as MTS 5. Although that study showed a higher proportion of patients in MTS 3 than that in this study, the low proportion of patients in MTS 5 was similar.

### Results of Ada and MTS

All patients were recruited from the ED waiting room, and the app provided advice to 56.3% (213/378) of cases to seek emergency treatment, as seen in Table 3. The app advised 39.4% (149/378) of the patients to see a GP and 4.2% (16/378) of the patients to make no physician appointment at all. The triage nurse assigned 19.8% (75/378) of patients as *urgent* (MTS 3; to be examined within 30 minutes), 75.9% (287/378) of the patients as *standard* (MTS 4; up to 90 minutes waiting time), and 4.2% (16/378) of patients as *nonurgent* (MTS 5; up to 120 minutes waiting time). To determine the safety of the app’s urgency assessment, the 2 systems were compared in Tables 3 and 4. Totally 91% (344/378) of patients were triaged the same or more conservatively when compared with the stand-alone MTS assessment, whereas 8.9% (34/378) of the patients were undertriaged. The chi-square test showed that the app’s urgency assessments’ distribution was equal in all examined departments (Cohen *d*=4.97; *P*=.05). Cohen *κ* calculated based on the merged comparison table showed low agreement between MTS and the app’s advice level (Cohen *κ*=0.033, 95% CI –0.023 to 0.089; weighted Cohen *κ*=0.035, 95% CI –0.630 to 0.700; prevalence-adjusted and bias-adjusted Cohen *κ*=–0.002, 95% CI –0.056 to 0.053; Table S4 in Multimedia Appendix 1).

Of 8.9% (34/378) of the undertriaged cases, 15% (5/34) were considered to be accurately triaged by all 3 panel physicians. The panel judged that for 26% (9/34) of the participants, the app’s urgency assessment could have posed a particular risk to the patient’s health when only considering the information the patient presented with, but when considering the whole case retrospectively, there was no risk. Of the 9 patients, 4 (44%) were considered to have received accurate advice by at least one physician.

**Table 3:** Overview of the urgency assessments by the two systems (rater 1: MTS^a^; rater 2: Ada; N=378) grouped in categories.

	MTS 3 (yellow), n (%)	MTS 4 (green), n (%)	MTS 5 (blue), n (%)	Total, n (%)
Call ambulance	23 (6.1)^b^	68 (17.9)^b^	5 (1.3)^b^	96 (25.4)
Emergency care	22 (5.8)^c^	91 (24.1)^b^	4 (1.1)^b^	117 (30.9)
Primary care within 4 hours	10 (2.6)^c^	20 (5.3)^b^	2 (0.5)^b^	32 (8.5)
Primary care within same day	10 (2.6)^d^	60 (15.9)^c^	3 (0.8)^b^	73 (19.3)
Primary care within 2 to 3 days	6 (1.6)^d^	34 (8.9)^c^	2 (0.5)^c^	42 (11.1)
Primary care within 2 to 3 weeks	2 (0.5)^d^	0 (0)^d^	0 (0)^c^	2 (0.5)
Self-care or pharmacy	2 (0.5)^d^	11 (2.9)^d^	0 (0)^c^	13 (3.4)
Self-care	0 (0)^d^	3 (0.8)^d^	0 (0)^c^	3 (0.8)
Total	75 (19.8)	287 (75.9)	16 (4.2)	378 (100)

^a^MTS: Manchester Triage System.

^b^Overtriage.

^c^Match.

^d^Undertriage.

**Table 4:** Urgency assessment results (N=378).

Description	Value, n (%)
App’s urgency assessments that matched with MTS^a^	128 (33.9)
App’s urgency assessments that were overtriaged in comparison with MTS	216 (57.1)
App’s urgency assessments that were undertriaged in comparison with MTS	34 (8.9)
App’s urgency assessments that were undertriaged in comparison with MTS but considered accurate by all panel physicians	5 (1.3)
App’s urgency assessments that were retrospectively not considered as an AHS^b^	9 (2.4)
Of the app’s urgency assessments that were retrospectively not considered as an AHS, the advices considered accurate by at least one physician	4 (1.1)
App’s urgency assessments that were considered as a potential AHS	20 (5.3)
Advice considered safe (all patients who were not considered to be in a potential AHS)	358 (94.7)

^a^MTS: Manchester Triage System.

^b^AHS: avoidable hazardous situation.

### Describing Potential AHS

In 5.3% (20/378) of the cases, at least one physician considered the app’s advice as potentially health-damaging if followed by the patient after considering all the case information. Of the 20 patients, 5 (25%) patients were admitted as inpatients, 10 (50%) patients were treated in the ED and subsequently discharged for further outpatient treatment, and 5 (25%) patients were discharged without treatment. The most common reason for attending the ED for these patients were wounds that needed to be stitched (5/20, 25%), followed by fractures (3/20, 15%), and infections (3/20, 15%). The list of potential AHS characteristics is presented in Table S5 in Multimedia Appendix 1**.** Fleiss *κ*, interpanel physician reliability of agreement when evaluating the likelihood of health risk, was Fleiss *κ*=0.0533 (95% CI –0.2267 to 0.3333), indicating slight agreement, following the interpretive guidelines reported by Fleiss et al [30].

## Discussion

### Principal Findings

Compared with usual hospital triage, 91% (344/378) of the participants were triaged identically or more conservatively by the app, and there was a *total undertriage* of 8.9% (34/378) of the participants, of which 59% (20/34) *were potential AHSs*. The app provided safe advice for 94.7% (358/378) of the patients when compared with the stand-alone MTS assessment, which served as the gold standard in this study. This includes identical or more conservative advice (344/378, 91%) and cases defined as safe by the physician panel (14/378, 3.7% no potential AHS). Of the 378 participants, 164 (43.4%) were not considered as emergency cases by the app.

### Degree of App Undertriage

The app’s rate of *undertriage* and rate of *leading to a potential AHS* are similar to those reported for telephone triage by HCPs. Placing this in context with the literature on triage, Meer et al [11] reported 4.6% (7/153) *potential AHS triage* (95% CI 1.85%-9.20%), Morreel et al [34] reported 17.01% (175/1029) undertriage for computer-assisted telephone triage, and Rørtveit et al [35] reported 10.8% (26/240) undertriage. In addition, Graversen et al [36] reported 17.7% (75/423) undertriage and specified 7.3% (31/423) *clinically relevant undertriage.* The urgency advice safety of the app is similar to or better than that reported in vignette studies of HCPs in a GP clinic setting, with 19.6% (69/352 vignette assessments) [37] and 17.1% (166/973 vignette assessments) undertriage for GP assistants [38]. In a recent vignettes study, GPs were compared with 8 SAAs, leading to a rate of undertriage of 13.74% (169/1230 vignette assessments) for GPs, with 2.92% (36/1230) of advice considered potentially unsafe [22]. This was compared with SAAs, including the Ada app, which reported a rate of 15% (30/200 vignette assessments) undertriage, with a 1.5% (3/200) rate of potentially unsafe advice (range for all SAAs 2.2%-20%).

Of the few studies reporting app-based self-assessment triage, a study reported 11.1% (14/126) undertriage [13] and another reported 5.2% (8/154) [39]. The latter was performed in a student health care center, exploring a different population with likely different presenting problems.

Examination of all collected information in this study enabled the identification of the reason for undertriage. Most commonly, the relevant condition was not modeled by the app; for example, 25% (5/20) of potential AHSs were related to non–life-threatening skin wounds, which must be examined and treated. Adapting the app to provide these scenarios would be a simple improvement. Potential AHSs also resulted from limitations in gathering information on previous injuries, a common reason for ED visits, as mild pain symptoms reported for the pre-existing injury site were not accompanied with descriptions of the injury itself, and therefore, received lower triage than appropriate after an accident. This can be resolved through an initial question about accidents. Multimorbidity also led to triage inaccuracy, as patients intermixed old and new symptoms and conditions.

### Degree of App Overtriage

The number of the apps *total overtriage* compared with nurse MTS triage was 57.1% (216/378), which compares with 42.1% (101/240) for intuitive triage of patients in ED by GPs [27], 19.3% (188/973 contacts) of patients in GP clinic by triage nurses studied through vignettes [38], 23.5% (101/430) for computerized triage decision support assisted nurses [36], and 55.8% (86/154) of patients for a prototype self-assessment triage system [39]. In the binary approach (call a physician or do not call a physician) of Verzantvorrt et al [13], a rate of 11.1% (14/126 home user) overtriage was reported, which is not comparable with an 8-point classification used in this study, in which the *total overtriage* includes even the most minor overtriage. The patient populations in the studies listed above partly differ from that in this study owing to a pretriage setting, making a direct comparison moderately difficult.

The *total overtriage* in this study was relatively high, as the app advises appropriately for the home setting. Although acknowledging that very conservative advice is undesirable, the approach of app manufacturers has been to reflect a *safety-first* approach [22,24,40]. This approach can be seen in a recently published comparison study of urgency assessments of 15 SAAs with those of laypersons, stating that SAAs classified a high number of low-urgency cases as emergencies (43/174, 24.7% vignettes), whereas true emergencies were detected in 80.6% (SD 17.9%) of cases [41]. The calculated ratio of overtriage to undertriage errors for SAAs was 3.5:1, showing the strong risk aversion of those apps with a number of overtriaged vignettes of 34.2% (182/532) and a range of accurate triage from 9% to 32%. This study falls in line with the studies mentioned above, showing the difficulty of web-based triage, and although a degree of overcautiousness is appropriate for safety, a balance should be maintained.

### Study Limitations and Strengths

A significant strength of this study is the variety of medical specialties included, as this provides a good representation of the average patient population in ED, in contrast to previous ED triage studies [42,43]. In addition, in contrast to vignette studies, the evaluation was directed prospectively, and patients performed the assessment by entering their data independently on their own. Moreover, unlike in previous studies [25], patients were included in the analysis of this study irrespective of whether the app’s medical knowledge modeled their diagnosed conditions.

To ensure that there was no delay in the treatment of patients with life-threatening conditions, the 2 highest categories of MTS were excluded. This also partially applies to patients who were triaged as *yellow* by MTS, who were often called by a nurse or student physician before recruitment, resulting in smaller proportion of these patients in the study.

A recognized challenge in comparison with triage methodologies is defining a reference standard [11]. Comparison of an innovative system against an established system is the most apparent validation approach; however, there are challenges. The MTS was created for patients in need of emergency treatment, who should all be examined by a physician within the same day, within a maximum waiting time of 120 minutes (German MTS) or 240 minutes (international MTS), even though not all patients who are presenting can be considered as patients with emergency [44,45]. However, the app was created for at-home use and has a broad spectrum of urgency advice gradations from *call ambulance* to *self-care*. Therefore, when creating the matching table between the MTS and the app, several app categories were equated with those of the MTS. Therefore, analysis using the widely used Cohen *κ* statistic could only be applied after merging the app’s urgency advice categories, where a low agreement between the raters was observed partly owing to the reasons listed above. For future studies, another statistical approach to measure the agreement between 2 raters (ie, triage approaches) should be developed.

In addition, the MTS, which was the system used as a gold standard in this study, has been shown in previous studies to have deficiencies affecting its overall safety and performance [46,47]. Specifically, it has been shown that the MTS has a high tendency to undertriage (range 11%-25%) and a low sensitivity for patients with high urgency; these were data that led to questioning the safety of the system. The rates for overtriage in the systemic review ranged from 7.6% to 54%, indicating potentially unnecessary resource use [46]. As only the undertriaged cases were individually assessed by the physician panel, all other results can only be considered as safe as the MTS itself. This underlines the importance of a good assessment and matching of urgency advice in future studies.

In the interpretation of these results, it should be considered that some of the authors of this paper were also affiliated with the company that developed the app.

In addition, the reported *total overtriage* is relatively high owing to a limitation in the study methodology. This could be resolved by having the appropriateness of all the app advices assessed by a panel instead of assessing only the patients who were undertriaged. Every patient in the ED, irrespective of whether he or she required ED treatment, received an MTS level requiring ED consultation. Studies have shown that the condition of 32%-37% of patients in the ED waiting room cannot be considered as urgent [37,38]. Therefore, we specified the matching criteria in the study planning phase, such that only MTS categories 1 to 3 were considered appropriate for patients in the ED. This led to the limitation that all patients categorized as MTS 4, who were advised by the app to see a GP within 4 hours or to go to the ED, were already considered as overtriaged according to the analysis plan. This included 47.4% (179/378) of patients, many of whom were likely appropriately triaged by the app and only classified as not matching owing to a limitation of the matching design in the analysis plan. However, it is recognized that owing to study design, as per definition, all patients who were classified as patients with emergency by the app could not be undertriaged as the MTS categories 1 and 2 were excluded from the study for reasons of safety and feasibility.

### Implications for Clinicians and Policy Makers and Future Research

It has been proposed that self-assessment triage apps could reduce unnecessary ED visits [18]. Of those patients who were assigned urgency advice in the lowest 3 MTS categories, only slightly more than half were considered as patients with emergency by the app, with 39.4% (149/378) of the patients being referred to a GP and 4.2% (16/378) of the patients being advised to not see a physician at all. This is a substantial number of patients who, by their own assessment, considered themselves as patients with emergency, but from what they stated in the app, possibly would have been comfortable with outpatient care. If these 43.4% (164/378) of patients could be redirected before their visit to the ED, this could lead to a substantial decrease in the number of patients in EDs.

The relatively high number of overtriaged cases in this study was, to a large degree, a result of the limitations of the study matching design, in which patients already waiting to seeing a GP in the next 4 hours were counted as overtriage. However, overtriage error of the app was detected in the context of this study. Pretriage has high potential to reduce the burden on EDs and to support the patient’s decision-making regarding where and when to best seek medical care before they visit the ED. It is important that developers of SAAs address the degree of overtriage by systems, while still ensuring that their system provides safe advice.

Further research in this area is needed to measure not only appropriate and safe advice in the at-home setting but also the willingness of patients to follow this. Studies should also address in more detail the appropriateness of advice of SAAs for users who require emergency treatment. We have identified several usability optimizations and recommendations for additions and optimizations of some ED-relevant presentations. These were incorporated into Ada’s product development process after reporting the study; for example, revising the advice level for ED cases that showed a high rate of overtriage.

### Conclusions

This observational study addressed an underresearched SAA triage topic in the ED [18]. We showed that the app provides urgency advice after patient *self-triage* that can be considered safe in 94.7% (358/378) of assessments when compared with the stand-alone MTS assessment, has a *rate of undertriage* and a *rate of triage with potential to be an AHS* equivalent to those of telephone triage by HCPs, and still, is a more conservative approach than direct ED triage by HCPs. In all, 43.4% (164/378) of patients who considered themselves as emergency cases were not considered so, indicating a possible relieve on EDs if the app was used at home. Continuous app optimization, followed by future research, should be conducted specifically in the at-home setting to investigate this hypothesis.

## Appendix

Multimedia Appendix 1 Additional study information and calculations.

Multimedia Appendix 2 Study data.

## Abbreviations

AHS: avoidable hazardous situation
ED: emergency department
GP: general practitioner
HCP: health care professional
MTS: Manchester Triage System
SAA: symptom assessment app
