# Supplementary material for: Safety of Triage Self-assessment Using a Symptom Assessment App for Walk-in Patients in the Emergency Care Setting: Observational Prospective Cross-sectional Study
Source: JMIR Mhealth Uhealth. 2022 Mar 28;10(3):e32340. doi: 10.2196/32340 (PMC9002590; doi:10.2196/32340)
Supplement: Multimedia Appendix 1 [file mhealth_v10i3e32340_app1.docx]

Multimedia Appendix

### **Multimedia** **Appendix, Table S1: STROBE Statement**

|  | **Item No** | **Recommendation** | **Page No** |
| --- | --- | --- | --- |
| **Title and abstract** | 1 | (*a*) Indicate the study’s design with a commonly used term in the title or the abstract | 1+2 |
|  |  | (*b*) Provide in the abstract an informative and balanced summary of what was done and what was found | 2+3 |
| **Introduction** | | | |
| Background/rationale | 2 | Explain the scientific background and rationale for the investigation being reported | 3+4 |
| Objectives | 3 | State specific objectives, including any prespecified hypotheses | 4 |
| **Methods** | | | |
| Study design | 4 | Present key elements of study design early in the paper | 5-7 |
| Setting | 5 | Describe the setting, locations, and relevant dates, including periods of recruitment, exposure, follow-up, and data collection | 4+5 |
| Participants | 6 | (*a*) Give the eligibility criteria, and the sources and methods of selection of participants. Describe methods of follow-up | 5 |
|  |  | (*b*) For matched studies, give matching criteria and number of exposed and unexposed | / |
| Variables | 7 | Clearly define all outcomes, exposures, predictors, potential confounders, and effect modifiers. Give diagnostic criteria, if applicable | 5-7 |
| Data sources/ measurement | 8* | For each variable of interest, give sources of data and details of methods of assessment (measurement). Describe comparability of assessment methods if there is more than one group | 5-7 |
| Bias | 9 | Describe any efforts to address potential sources of bias | 5 |
| Study size | 10 | Explain how the study size was arrived at | 5, 7+8 |
| Quantitative variables | 11 | Explain how quantitative variables were handled in the analyses. If applicable, describe which groupings were chosen and why | 6+7 |
| Statistical methods | 12 | (*a*) Describe all statistical methods, including those used to control for confounding | 6+7 |
|  |  | (*b*) Describe any methods used to examine subgroups and interactions | 6+7 |
|  |  | (*c*) Explain how missing data were addressed | 6+7 |
|  |  | (*d*) If applicable, explain how loss to follow-up was addressed | / |
|  |  | (*e*) Describe any sensitivity analyses | / |
| **Results** | | |  |
| Participants | 13* | (a) Report numbers of individuals at each stage of study—eg numbers potentially eligible, examined for eligibility, confirmed eligible, included in the study, completing follow-up, and analysed | 7+8 |
|  |  | (b) Give reasons for non-participation at each stage | 7+8 |
|  |  | (c) Consider use of a flow diagram | 8 |
| Descriptive data | 14* | (a) Give characteristics of study participants (eg demographic, clinical, social) and information on exposures and potential confounders | 8 |
|  |  | (b) Indicate number of participants with missing data for each variable of interest | 8 |
|  |  | (c) Summarise follow-up time (eg, average and total amount) | / |
| Outcome data | 15* | Report numbers of outcome events or summary measures over time | 8+9 |

###

### **Multimedia** **Appendix, Table S2: Merged urgency assessments by the two systems (Rater 1: MTS, Rater 2: Ada)**

|  | **MTS 3 (yellow)** | **MTS 4 (green)** | **MTS 5 (blue)** |
| --- | --- | --- | --- |
| **Emergency Care, Primary Care 4 hours** | 0 (match) | 4 (Undertriage) | 8 (Undertriage) |
| **Primary Care same Day** | 1 (Overtriage) | 0 (match) | 4 (Undertriage) |
| **Primary Care 2 to 3 Days**  **Primary care 2 to 3 weeks**  **Self-Care Pharma**  **Self-Care** | 2 (Overtriage) | 1 (Overtriage) | 0 (match) |

###

### **Multimedia** **Appendix, Table S3: Decisions of panel members on undertriaged case**

| **N** | **PA** | **AA** | **DA** | **RA** | **Physician 1 Explanation** | **Physician 2 Explanation** | **Physician 3 Explanation** | **reason given at online discussion for calling it as "potentially health damaging"** |
| --- | --- | --- | --- | --- | --- | --- | --- | --- |
| **5** | AHS | 6 | 3, 4, 5 | c, c, c | Taking into account the Wells score (which is unfortunately not completely queried in the anamnesis), a deep vein thrombosis must be excluded promptly. Note: Acute lung embolism. It could have been detrimental to health. | A new onset of unilateral leg swelling should be clarified within a few days, even if no thrombosis/infection was present. | Comes already susp. thrombosis. Anticoagulation important, acute lung embolism therefore possible. Presentation same day. | The patient had a suspected lung embolism and needed to be checked and treated on the same day. The patient did not have a lung embolism (that was the result of ED diagnostics) and was send back home without treatment. The reason for calling this an AHS was, that the patient had to have a reason for his problem which should be checked in a shorter period of time than the 2-3 weeks adviced by the App. |
| **31** | AHS | 5 | 3, 4, 4 | c, b, c | High-fever bacterial infection that should be treated promptly with antibiotics to avert a septic course. Additional risk of exsiccosis and electrolyte disturbance in case of insufficient fluid intake | In synopsis indicative of pyelonephritis, strong indication for antimicrobial treatment, however, if delayed by 1-2 days, life-threatening consequences/permanent damage unlikely. | Fever, chills, flank pain already points in the direction of urinary tract infection | Patient had a severe urinary tract infection and had to be treated in-patient with antibiotics and fluids. |
| **54** | Appropriate | 5 | 4, 4, 3 | a, a, b | In case of pronounced pain symptoms and lack of improvement after taking p.o. analgesics, however, a medical presentation should be made on the same day. | Migraine limits quality of life but is not threatening; severity of symptoms and failure of self-treatment warrants same-day medical presentation | Question 1: Very delicate. With migraine, yes, the case went well. Anamnestically, however, the app must know; migraines known, well adjusted. Sudden (within a day is indicated), as well as the pain character must be worked out exactly (otherwise rather level2). Differential diagnosis to SAB.  Eye pain (described in singular according to app) and age also suggest initial diagnosis of multiple sclerosis. Ex ante view! |  |
| **58** | AHS | 5 | 2, 2, 3 | c, d, c | Rapid weight gain, dysuric complaints, increased retention values and pathological bleeding tendency in the case of a known C2-abuse require a prompt clarification, especially of the cardiac involvement, the coagulation situation and electrolyte status (cardiac arrhythmias). | Potentially life-threatening decompensated combination of liver cirrhosis and heart failure --> Emergency | Still compensated in triage because there is no respiratory distress (negated). If there is respiratory distress, it quickly jumps to 2 or 1.(Decompensation). As a sign of rapid progression and incipient decompensation, rapid weight gain is a clue. | Patient had a probably decompensated liver and cardiac cirrhosis which had to be compensated in-patient |
| **60** | Appropriate | **4** | 2, **4, 4** | b, a, b | Prompt diagnosis needed, especially , whether it is a dislocated fracture or not - prompt therapy to minimize complications in the course.  It could have been detrimental to health but not life threatening - difficult to decide between b or c. | With medical care on the same day, no damage is expected, thus appropriate advice | Same triage. An uncomplicated distal radius fracture on the same day does not cause any problems (the important thing is whether there is an abrasion over the fracture). |  |
| **66** | Retrospective no AHS | **4** | 2, **5, 4** | c, a, a | Answer c- only by clinical , neurological examination could functional paraparesis be diagnosed.  E.g., Guillian-Barre syndrome had to be excluded, which could be life-threatening if not recognized. | Diffuse/elusive complaints ultimately based on pre-existing conditions. | Equally triaged. |  |
| **78** | AHS | 5 | 2, 3, 2 | c, c, d | Patient is under oral anticoagulation- persistent bleeding, therefore prompt medical presentation with suture closure necessary. Tetanus protection must also be obtained promptly. A health damage after two-three days can therefore not be excluded. | Wound care with suture, should be done within hours, in case of secondary wound healing possible functional damage, check of tetanus protection indicated on the same day, possible consequences are life threatening. | Trauma was not inquired. Tingling in the N. medianus area, could also be a nerve involved.nurse bescheribt contusion wound! | Patient had an incised wound, needs to be stitched in the ER and possibly vaccinated |
| **87** | AHS | 4 | 2, 3, 2 | c, c, d | Prompt surgical treatment to minimize complications in the course. I consider a life-threatening situation to be rather unlikely - but I differentiate between a threat to life and damage to health, hence answer c. | Care with suture should be provided within hours, adverse cosmetic consequences likely with secondary wound healing, tetanus protection should be checked the same day | There is a very clear report of a fall on the ear, with vulnerable cartilage behind it. | Patient had a tear of his ear lobe which needed immediate surgical care |
| **89** | AHS | 7 | 5, 5, 5 | a, c, ? | Unfortunately, the Ada report does not explicitly state the exact period over which the symptoms (constipation) persist.  I consider 1-7 days to be too imprecise for these symptoms. | Constipation lasting several days and blood accumulation should be clarified by a physician. | There could be a subileus behind it. Questions such as "winds come off," "have you had it before," "nausea," and "vomiting" were missing. I consider SelfPharma, i.e. taking medication from the pharmacy without contacting a doctor for abdominal pain, to be dangerous. | A patient with several days of obstipation and blood on stool should be at least seen by a GP |
| **103** | Retrospective no AHS | 7 | 1, 1, 1 | c, c, d | Answer 1c. Patients after an accident with a present accident mechanism need immediate medical clarification.  Only after clinical and radiographic examination, a fracture in the cervical spine area and also secondary injuries could be excluded. | Due to the mechanism of the accident, the current guideline on polytrauma provides for a shock room indication. | Polytrauma criteria met |  |
| **109** | AHS | 7 | 4, 3, 3 | c, d, c | Answer c. Acute prepatellar bursitis with high levels of infection, inadequate antibiotic therapy, and progression of clinical signs of inflammation should be presented promptly to a physician. | The constellation suggests bacterial bursitis with failure of antimicrobial therapy. There is an indication for surgical therapy. | The history elicits a classic bursitis that does not respond to antibiotics. | Patient was sent to the ER by an orthopaedic doctor for a surgical treatment of his severe knee infection |
| **124** | Retrospective no AHS | 8 | 2, 3, 4 | c, c, c | Answer c. Cranial trauma with persistent symptoms (cephalgia) should be promptly clarified by a doctor - check for intracranial bleeding or facial skull fracture. | Due to the mechanism serious injury conceivable. | Ada omits trauma and questions about anticoagulation. Midface fracture possible. And possibly more if anticoagulation is still taken. |  |
| **125** | AHS | 7 | 3, 4, 4 | c, b, d | Answer c. Increasing pain symptoms and immobility ("could no longer leave the bed independently") should lead to a prompt medical presentation, especially since there was a history of a fall 4 weeks ago with a pelvic fracture. A renewed imaging was necessary, to exclude further fractures. | Osteoporosis is likely. In the context, acute back pain should be considered indicative of a fracture. Medical clarification required. | Fresh lower spine fracture, needs control and X-ray to make sure the fracture does not re-sinter. | patient had a fractured vertebral body which is a severe condition that needs to be checked in the ED |
| **141** | Appropriate | **4** | **4, 4, 4** | a, a, a | Answer 1a- same-day medical presentation, for initiation of antibiotic therapy for highly suspected bacterial disease. | Ada's advice is appropriate and sufficient. | Equal triaging |  |
| **143** | Retrospective no AHS | 7 | 4, 4, 4 | c, b, c | Answer 1c- Exclude fracture only after medical and radiographic examination. | Due to the injury, the tetanus vaccination should be checked on the same day and refreshed if necessary. Due to the mechanism of the accident, a medical examination on the same day is advisable. | Radiological fracture exclusion |  |
| **155** | AHS | 7 | 4, 5, 5 | c, a, b | Answer 1c (harmful to health). Increasing immobility with knee trauma- medical clarification necessary and prophylactic administration of a heparin to prevent deep vein thrombosis. | I consider a medical examination to be reasonable in view of the accident mechanism. However, adverse consequences/permanent damage are not to be expected in the constellation due to delayed diagnostics. | Knee contusion as a diagnosis for knee twisting does not exist. Since BG case must refer it to a D-doctor. | Patient had a twisted knee, needed thromboprophylaxis pain medication and imaging. Could have also gone to an orthopaedic on the same day. |
| **193** | AHS | 8 | 2, 3, 2 | c, c, d | Head injuries with persistent cephalgia and dizziness should be examined by a physician for assessment. It is possible that 1c could have resulted. | Due to the wound, a medical examination should be performed within hours in order to perform adequate wound care and skin suturing, which was probably indicated here. Thus, a less favorable cosmetic result is now to be expected. Due to commotio and to check the tetanus vaccination protection, a medical examination should be performed on the same day. | Gaping wound/wound care cause? Tetanus? Adadiagnoses b and c contradict Selfcare ;-) | Patient had an incised wound, needs to be stitched in the ER and possibly vaccinated |
| **206** | Retrospective no AHS | 7 | 1, 2, 4 | c, c, b | 1c. Cervical spine trauma after rear-end collision should be medically clarified immediately. Only after a detailed anamnesis and clarification of the course of the accident and the clinical examination could a health impairment be ruled out. | Due to the accident mechanism (according to the guideline polytrauma shock room indication), a medical examination should take place immediately. | No head or neck injury raised. No floated airbags mentioned (unusual, airbags deploy from 30 km h). |  |
| **207** | AHS | 5 | 3, 3, 2 | b, c, d | Bleeding intensity and tetanus protection were not queried via Ada. | There is an indication for wound care with suturing within hours. Risk of an unfavorable cosmetic and/or functional result. Tetanus protection must be checked on the same day. | Open wound and then also over joint. Disaster. | Patient had an incised wound, needs to be stitched in the ER and possibly vaccinated |
| **208** | AHS | 7 | 4, 4, 3 | b, c, b | Answer 1b, where Ada does not consider the accident mechanism. | Wound care, medical examination and verification of tetanus protection should be done on the day of the accident. | Both doctor and app do not query for thorax and abdomen! Can also go out blse. Not in this case. Personal note: I have already seen 2 life-threatening splenic ruptures because of bicycle handlebars into the abdomen. Here, that would be a diagnostic elicitation error (not a diagnostic elicitation error). So grossly negligent! | Patient had a car crash, needs to be seen in the ER for wound treatment and check up |
| **210** | AHS | 7 | 4, 2, 2 | c, d, d | Newly appeared paresis must be clarified by a physician.Permanent damage is to be expected with non-operative treatment. Answer c. | A herniated disc with motor deficit is an emergency! | Paraplegia danger | Patient had a disc prolaps and needed surgical treatment |
| **245** | Appropriate | **4** | 5, **4, 4** | a, a, b | With mild symptoms, one could have waited - DD:viral genesis | Ada's advice was absolutely sufficient. There was a transfer triage in the ZNA | Same triaging. No fever. |  |
| **253** | AHS | 4 | 2, 2, 2 | c, b, d | 1c. Clinical: Fall on the face and acute onset of hearing loss require a  prompt medical examination and imaging to rule out serious injuries that could then have been life-threatening. | Accident mechanism and occurred injury constitute an emergency. If Ada's advice had been followed, only a delay of hours would have occurred. | Fracture, possible rupture of eardrum (difficulty hearing, lack of pressure equalization in conjunction with TRAUMA. | Patient had a fracture of his auditory canal and several contusions on his face → needed reposition in the ER, could not wait the whole day (Ada said PrimaryCareSameDay) |
| **254** | Retrospective no AHS | **4** | **4, 4,** 2 | b, a, c | 1b- previous physician contact had taken place ( referral via pulmonology outpatient clinic). Moderate symptoms. | Same day supply is appropriate. | The nurse referral diagnosis is critical here. It is a physician to physician referral for V. a. renal failure. |  |
| **258** | Retrospective no AHS | 7 | 4, 4, 4 | b, c, c | Radiographic diagnosis was necessary to rule out atypical pneumonia, but I would nevertheless  Take answer 1b. | Same-day examination indicated to clarify suspected pneumonia. | Exclusion of pneumonia, chills do not fit intercostal neuralgia. Fever negated. If it had been pneumonia, that would not be good. |  |
| **274** | Retrospective no AHS | **4** | **4**, 2, 3 | b, a, d | No explanation | A motor deficit, and bladder and rectal dysfunction are emergencies per se. Additional damage is not expected if Ada's advice is followed. | Pat has unrecognized urinary incontinence, not queried in Ada, also missing a stool query. |  |
| **283** | Retrospective no AHS | **6** | 5, **6,** 4 | b, a, d | No explanation | The advice is appropriate in the specific situation. | With the history, the susp. neuroborreliosis results. |  |
| **291** | AHS | 4 | 1, 1, 2 | c, c, c | 1c- Indication of pectanginal symptoms, as well as dyspnea on exertion and palpitations. Diabetes and hypertension in the previous diagnoses - immediate diagnosis necessary. | The patient is considered critically ill: tachyarrhythmia absoluta with decompensation, lactate, chest tightness. | Decompensation | Patient was cardially decompensated, needed treatment immediately and was admitted to the Chest pain unit |
| **314** | AHS | 8 | 4, 5, 4 | c, b, ? | New onset of positional paresthesias in the upper extremity, state after polytrauma requires repeat imaging and medical presentation. It could possibly have been harmful to health.Answer C. | Rather diffuse symptoms. I consider a medical examination within days to be indicated, especially in view of the sensory disturbance. | SelfCare? With the history and the clinic? | Patient had parasthesia after polytrauma, needs to be at least seen by a physician |
| **326** | AHS | 5 | 2, 2, 2 | c, c, d | Prompt clarification with the poison control center necessary - unclear toxic cleaner - could have led to further burns (especially problematic in the facial area). A high degree of skin damage could only be ruled out in the course. Therefore answer c. | Emergency examination indicated. High potential for damage if examination is delayed. | Noxe (resorption?), skin damage | Patient was in contact with a poisonous substance, needed to be checked by a physician immediately |
| **342** | Appropriate | **4** | **4, 4**,1 | b, a, ? | Answer b. Gait disorder already known- no acute focal neurological deficits- symptoms regressed under stress. | Since symptoms have been present for several days, same-day examination is appropriate. | Could have been a stroke Unfortunately, not well differentiated by ADA. |  |
| **372** | AHS | 7 | 3, 4, 3 | c, d, d | Answer c. An infected hematoma after i.m. injection must be diagnosed and treated promptly. Risk of sepsis and local complications. | Same day examination/treatment indicated due to abscess. | The patient's file shows the classic signs of suspected infection. Swelling, redness, hyperthermia. Trauma (injection in this case) not inquired. | Patient had an infected hematoma which needed surgical treatment |
| **376** | AHS | 4 | 2, 2, 2 | c, d, d | A gaping cut should be examined promptly by a doctor to rule out the possibility that deeper structures (e.g. tendons) have also been injured. Only then can permanent damage be ruled out.  Therefore answer c. | The depth of the injury should prompt immediate examination/care. Injury to nerve/vessel conceivable. Treatment with suture indicated. Tetanus protection must be checked. | Cut, infection, joint involvement possible | Patient had an incised wound, needs to be stitched in the ER and possibly vaccinated |
| **395** | AHS | 7 | 5, 4, 4 | a, d, d | Answer a.  Imaging diagnostics performed in advance. Patient very confused and persistent pain symptoms, therefore medical presentation for discussion of findings is advisable. | Relevant injury with potential for high permanent damage. Medical treatment required. | Comes to the ZNA after a week, fracture exclusion is important. Without the CT, everything can be damaging. | Patient had several fractures after a fight, needed to be checked |

N=Number, PA = Panel Assessment, AAL= Adas Advice Level, DAL= Doctors Advice Level, RA = Risk assessment

Risk assessment letters:

A = Unlikely to be life-threatening/damaging to health

B = rather unlikely to be life-threatening/damaging to health

C = rather likely to be life-threatening/damaging to health

D = likely to be life-threatening/damaging to health

### **Multimedia** **Appendix, Table S4: Results on merged urgency assessments by the MTS and the app, (Rater 1: MTS, Rater 2: Ada)**

|  | **MTS 3 (yellow)** | **MTS 4 (green)** | **MTS 5 (blue)** | **Sum** |
| --- | --- | --- | --- | --- |
| **Emergency care**  **Primary care 4 hours** | **32** | 113 | 6 | 151 |
| **Primary care same day** | 10 | **61** | 3 | 74 |
| **Primary care 2 to 3 days**  **Primary care 2 to 3 weeks**  **Self-care pharma**  **Self-care** | 11 | 48 | **2** | **61** |
| **Sum** | 53 | 222 | **11** | **286** |

Cohen’s kappa: 0.033 (95%-CI: -0.023, 0.089), Weighed Cohen’s kappa: 0.035 (95%-CI: (-0.630, 0.700), prevalence-adjusted and bias-adjusted kappa: -0,002 (95%-CI: (-0.056, 0.053))

###

### **Multimedia** **Appendix, Table S5: Potential AHS Patients characteristics**

| **N** | **Age** | **S** | **MTS** | **AAL** | **Dep** | **Presenting Symptoms** | **Comment nurse** | **Treatment in ED** | **further procedure** | **Diagnosis** |
| --- | --- | --- | --- | --- | --- | --- | --- | --- | --- | --- |
| **5** | 55 | m | 3 | 6 | O | swollen Knee, one week until one month | suspected thrombosis, pain lower extremity | - | send home without treatment | no discharge diagnosis (exclusion lung embolism) |
| **31** | 46 | m | 3 | 5 | N | staggering vertigo(longer than a year) and headache (longer than a year, both sides, throbbing, moderate intensity), vomiting (one week until one month) | neurology, vertigo - staggering vertigo, nausea | antibiosis and fluid substitution | in patient admission | infection (urinary tract) |
| **58** | 56 | m | 3 | 5 | I | rapid weight gain, frequent need to urinate (one week to one month), more intense urge to urinate( one week to one month) | Unknown - renal failure, liver cirrhosis, abnormal cardiac history. | Inpatient admission for recompensation, ascites puncture, pleural puncture, palliative therapy adjustment for end-stage heart failure. | in patient admission | Decompensated liver and cardiac cirrhosis (suspected cardiorenal syndrome) |
| **78** | 68 | m | 3 | 5 | O | finger pain (shorter than one day; no effect with movement; one side; moderate pain) | Orthopedics/Trauma, upper extremity injury / contusion wound middle finger left | wound cleansing and closure with 4 sutures, analgesics, discharge to outpatient follow-up treatment | send home after treatment | wound (finger) |
| **87** | 68 | m | 3 | 4 | ENT | Earache (shorter than one day, one ear, mild intensity) | Unknown cause of injury / Injury to left ear, condition after fall | outpatient surgical treatment | in patient admission | wound (ear) |
| **89** | 22 | m | 4 | 7 | I | Digestive problems: one day to one week | Gastrointestinal, constipation / currently constipation | clysms and macrogel administration | send home after treatment | no discharge diagnosis |
| **109** | 61 | m | 4 | 7 | O | Knee pain(1-7 days, improving with activity, one side, moderate pain ), swelling of the leg ( 1-7 days, one side) | Orthopedics/Trauma, pain lower extremity - acute bursitis right knee, inflammation levels high, lab attached, anitbiotics | - | send home without treatment | infection (knee) |
| **125** | 61 | m | 4 | 7 | O | Lower back pain (one day to one week, worsening with exercise, moderate pain, not radiating to the leg) | Orthopedics/Trauma, back pain - back pain lumbar spine area, condition after fall 3 weeks ago | - | send home without treatment | fracture (lower back) |
| **155** | 24 | m | 4 | 7 | O | Knee pain (one day to one week, no effect with activity, one side, moderate intensity) | Orthopedics/Trauma, lower extremity pain, right knee pain, twisted yesterday | prescription Clexane, forearm support, pain medication, MRI referral | send home after treatment | twisted knee |
| **193** | 19 | m | 4 | 8 | N | Sore on the scalp (shorter than one day, painful, not itchy, crusted surface, no black scab), Headache (shorter than one day, both sides, throbbing, intensifying when bending forward, mild intensity), dizziness (horter than one day, intensifying when standing up abruptly, like a feeling of lightheadedness) | Neurological, head injury / small laceration on the head, slight dizziness, condition after collision | wound care | send home after treatment | wound (head) |
| **207** | 43 | m | 3 | 5 | O | Finger skin wound | Orthopedics/Trauma, upper extremity injury/cut to left small finger BG | Cleaning, stitching with 3 stitches, tetanus refreshed | send home after treatment | wound (finger) |
| **208** | 20 | f | 4 | 7 | O | Knee pain (less than one day, worsening with activity, laterality both sides, mild intensity), foot pain (less than one day, worsening with activity, one side affected, mild intensity) | Severe trauma - blunt, condition after car accident, swelling right knee, swelling left under arm, abrasion left ankle | wound care | send home after treatment | car accident (Abrasion outer ankle left, bruise and hematoma left lower leg, bruise right kneecap) |
| **210** | 22 | m | 4 | 7 | O | Reduced mobility of the shoulder (one week to one month) | Orthopedic/Trauma upper extremity pain/neuralgic amyotrophy right, high grade arm plexus palsy right side for 2 weeks | inpatient: Surgical treatment with cage insert | in patient admission | disc prolapse |
| **253** | 35 | f | 3 | 4 | ENT | Fullness in the ear( shorter than one day, one ear), bruising on the face (shorter than one day) | ENT (mouth, throat, neck), trauma face / fell on face, hematoma chin, hearing loss on left ear | Reduction under local anesthesia on the left with strip insertion | send home after treatment | fracture (anterior external auditory canal), bruise and hematoma (chin) |
| **291** | 65 | m | 3 | 4 | I | Decreased physical performance (longer than one year), dizziness (longer than one year, aggravated by abrupt rising, like swaying) | Cardiovascular, general weakness / announced by GP via phone, beginning cardiac decomp. | Administration of 10mg Velox as a frequency control trial, i.v. administration of 2g magnesium, uptake on Chest-Pain-Unit | in patient admission | Decompensated cardiac cirrhosis, persistent atrial fibrillation |
| **314** | 66 | m | 4 | 8 | N | Tingling in one part of the body (one week to one month, both sides) | Orthopedics/Trauma, pain in upper extremity / new onset of position-dependent paresthesia in arms after polytrauma, especially subluxation of the rib heads for approx. 2 weeks | - | send home without treatment | Unclear paresthesia, suspected costochondritis |
| **326** | 67 | m | 3 | 5 | D | Pain in the face (shorter than one day) | Skin, other skin problems / injury with substance on the right cheek | Contact poison control center, recommend regular flushing | send home after treatment | contact to poisonous substance |
| **372** | 52 | f | 4 | 7 | O | Bruise on the thigh (one day to one week) | Unknown / Consil | after puncture indication for surgical treatment | send home after treatment | infection (upper leg) |
| **376** | 33 | f | 4 | 7 | O | Skin wound on the finger (shorter than one day, not painful, not itchy, no crusted surface, black scab) | Orthopedics/Trauma, injury upper extremity, cut finger left hand | wound care | send home after treatment | wound (finger) |
| **395** | 25 | f | 4 | 7 | OMS | Pain in the sinus (one day to one week) | Unknown, Condition after brawl, suspected zygomatic and maxillary fracture | Clarification of probable persistent hypesthesia, no treatment | send home without treatment | fracture (zygomatic and maxillary) |

N=Number, S=Sex (m=male, f=female), Dep=Departmemt (O=Orthopaedics, N=Neurology, I=Internal Medicine, D=Dermatology, ENT= Ear Nose Throat, OMS= Oral and Maxillofacial Surgery), MTS = Manchester Triage System, AAL= Adas Advice Level

### **Multimedia** **Appendix, Table S6: Predefined matching of urgency assessments by the two systems (Rater 1: MTS, Rater 2: Ada)**

|  | **MTS 3 (yellow)** | **MTS 4 (green)** | **MTS 5 (blue)** |
| --- | --- | --- | --- |
| **Call Ambulance** | Overtriage | Overtriage | Overtriage |
| **Emergency Care** | Match | Overtriage | Overtriage |
| **Primary Care 4 Hours** | Match | Overtriage | Overtriage |
| **Primary Care Same Day** | Undertriage | Match | Overtriage |
| **Primary Care 2 to 3 Days** | Undertriage | Match | Match |
| **Primary Care 2 to 3 Weeks** | Undertriage | Undertriage | Match |
| **Selfcare Pharma** | Undertriage | Undertriage | Match |
| **Selfcare** | Undertriage | Undertriage | Match |

The color coding is intended to highlight the assignment to the three categories "Match" "Overtriage" and "Undertriage".

**Multimedia** **Appendix, Table S7: Age distribution and mapping of urgency advice**

|  | **18-29** | **30-39** | **40-49** | **50-59** | **60-69** | **70-79** | **80-89** | **90-99** |
| --- | --- | --- | --- | --- | --- | --- | --- | --- |
| **In total** | 92 | 59 | 56 | 71 | 54 | 27 | 6 | 1 |
| **Match** | 31 | 22 | 16 | 26 | 17 | 8 | 2 | 0 |
| **Over-triage** | 49 | 31 | 37 | 41 | 28 | 19 | 4 | 1 |
| **Under-triage** | 12 | 6 | 2 | 4 | 9 | 0 | 0 | 0 |


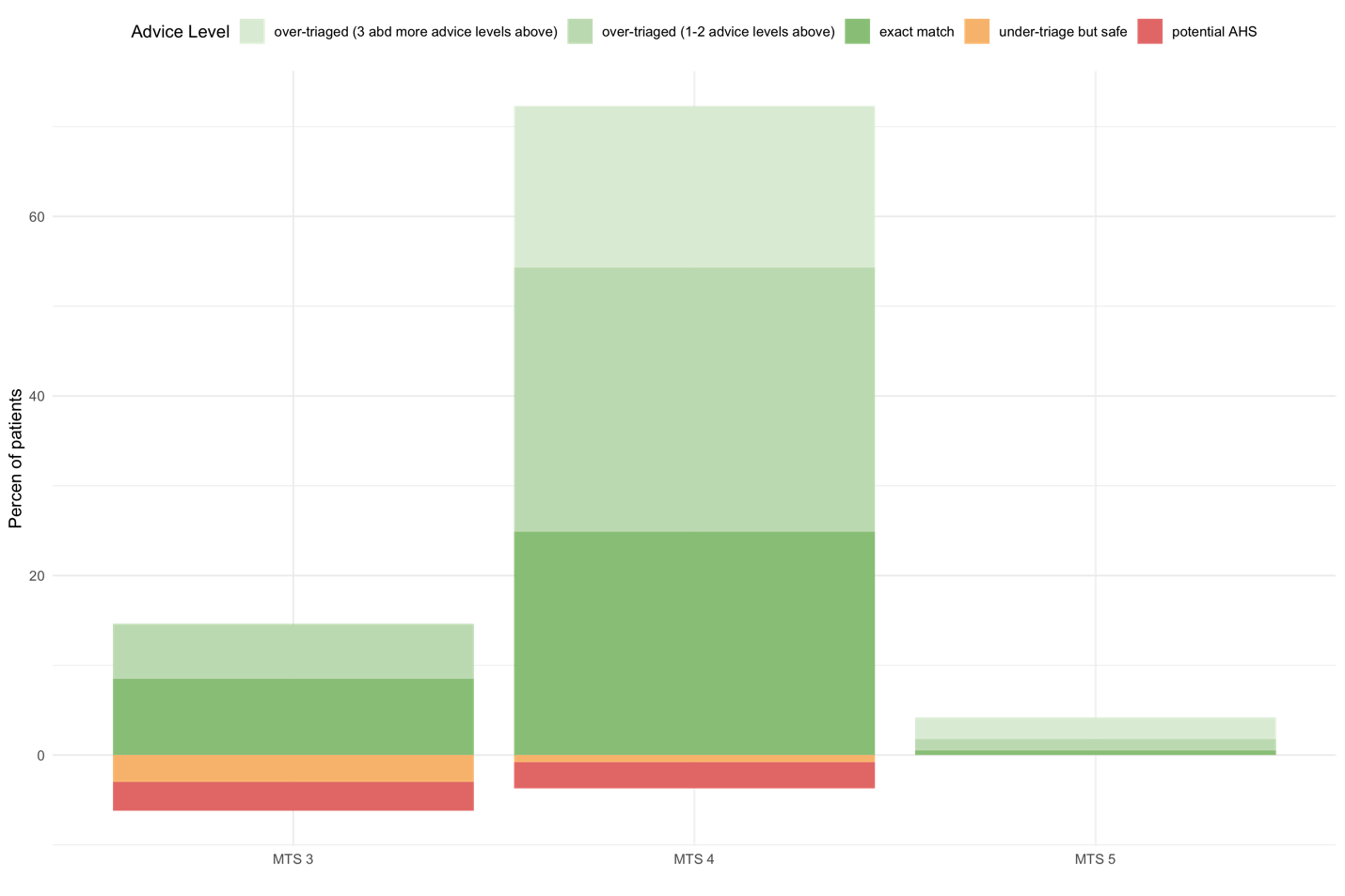


**Multimedia Appendix, Figure S1: Comparison of urgency advice by MTS and Ada**
